# Reviewer List 2023

**DOI:** 10.1093/tas/txae004

**Published:** 2024-02-10

The Section Editors and the Editor-In-Chief of *Translational Animal Science* like to thank the many scientists who have contributed their time and talents reviewing manuscripts for the journal. Reviewing for the journal is critical to ensuring that the best science is published. The expertise of these individuals is invaluable to the journal, the animal science community and to those that depend on the high-quality science that is published.

## Top reviewers (3+ reviews completed)

Alves Correa Carvalho da Silva, Felipe

Bradley, Casey

Cappellozza, Bruno

Carr, Chad

Carvalho, Pedro

Ellis, Mike

Engle, Terry

Ferraretto, Luiz

Goodband, Robert

Gouvea, Vinicius

Hansen, Stephanie

Johnson, Jay

Kiarie, Elijah

Lalman, David

Oltjen, James

Posbergh, Christian

Ranches, Juliana

Relling, Alejandro

Samuelson, Kendall

Schoonmaker, Jon

Smith, Zachary

Vyas, Diwakar

Yu, Haipeng

## Reviewers

Adegbeye, Moyosore

Ahola, Jason

Akit, Henny

Alagbe, Emmanuel

Alexander, Brenda

Alhidary, Ibrahim

Alugongo, Gibson M

Alvarez Garcia, Wuesley Yusmein

Andreini, Emily

Arentson, Roger

Arnandes, Renata

Arthington, John

Ashley, Ryan

Aymerich, Pau

Azain, Michael

Baker, Hannah

Baldassini, Welder

Bass, Benjamin

Batistel, Fernanda

Beck, Paul

Becker, Spenser L.

Berg, Kiah

Bernardes, Thiago

Beyer, Erin

Bible, Megan

Blatchford, R. A.

Bohrer, Benjamin

Boler, Dustin

Boler, Dustin D.

Bormann, Jennifer

Brauner, C.

Broomhead, Jonathan

Burkey, Tom

Cabral, Luciano

Callaway, Todd

Cannas, Antonello

Carakostas, Michael

Cardoso, Fabiana

Carstens, Gordon

Cassens, Drew

Castilha, Leandro Dalcin

Cedeño, Yolanda

Cemin, Henrique

Chase, Chris

Chen, Chi

Chen, Jun

Chen, Sean

Chiba, Lee

Chizzotti, Mario

Cleere, Jason

Cole, N.

Colle, Michael

Crenshaw, Joe

Cross, Amanda

Da Silva, Aleksandro

Dahlen, Carl

Dalton, Joseph

Daniel, Jay

De Girolamo, Paolo

Dee, Scott A.

DeRouchey, Joel

DiCostanzo, Alfredo

Dilger, Anna

DiLorenzo, Nicolas

Distl, Ottmar

Drewery, Merritt

Duttlinger, Alan

Ealy, Alan

Edwards-Callaway, Lily

El-Zaiat, H. M.

Enjalbert, Francis

Erickson, Mary

Espinosa, Charmaine

Faciola, Antonio

Falowo, Andrew

Fan, Peixin

Felix, Tara

Feoli, Carolina

Flowers, William

Foote, Andrew

Foster, Jamie

Fraga, Alícia

Fraile, Lorenzo

Froehlich, Kelly

Fushai, Felix

Gabler, Nicholas

Gadberry, Shane

Gaines, Aaron

Gebhardt, Jordan

Gifford, Craig

Gindri, Marcelo

Glaze, Benton

Gonzalez, John

Grandin, Temple

Greiner, Laura

Hackman, Tim

Hamdon, Hatem. A.

Harrison, Meredith

Harvey, Kelsey

Herring, Andy

Hoseinifar, Seyed Hossein

Hosseindoust, Abdolreza

Huber, Lee-Anne

Jang, Young Dal

Jennings, Jenny

Johnson, Greg

Jones, Helen

Kamboh, Asghar

Kamphues, Josef

Karisch, Brandi

Kegley, Elizabeth

Kim, In Ho

Kim, Jonkyoo

Kim, Kwangwook

Kim, Sung Woo

Kleinschmit, Daryl

Klopatek, Sarah

Lancaster, Phillip

Landy, Nasir

Lauridsen, Charlotte

Lavergne, Theresia

Leal, Leonel

Lents, Clay

Lerner, Annie

Létourneau-Montminy, Marie-Pierre

Levesque, Crystal

Li, Qingyun

Lindemann, Merlin

Lonergan, Steven

Lu, Zeqing

Mafi, Gretchen

Marçal, Danilo

Marques, Rodrigo

Martins, Thiago

Mastellar, Sara L.

McPhee, Malcolm

Medeiros da Silva, Gleise

Menchaca, Alejo

Menegat, Mariana

Menendez, Hector

Mercadante, Vitor R. G.

Metz, Mickie

Metzler-Zebeli, Barbara

Meyer, Allison

Meyer, Robert

Miao, Jinfeng

Miller, Phillip

Morota, Gota

Morris, Cheryl

Mosley, Jeff

Mullenix, Mary

Mulliniks, Travis

Nagaraja, TG

Narayan, Edward J.

Nielsen, Brian

Nogueira, Ériklis

Nonneman, Dan

Nwachukwu, Chinwe

Obanla, Lukmon

Oloruntola, Olugbenga

Olson, KC

Osowe, Clement

Overton, Thomas

Pace, Lanny

Paganoni, B. L.

Patra, Amlan

Patra, Amlan Kumar

Paula, Eduardo

Paulk, Chad

Penner, Gregory

Pereira, Angélica

Perez-Palencia, Jorge

Pinedo, P

Pitesky, Maurice

Place, Sara

Poole, Daniel

Prestegaard-Wilson, Jacquelyn

Ramirez, Brett

Richert, Brian

Risso, Analia

Robison, Jon

Rocha, Cecilia

Rosero, David

Rouquette, Monte

Rouzbehan, Yousef

Salak-Johnson, Janeen

Samuel, Ryan

Scheffler, Jason

Schinckel, Allan

Schmidt, Ty

Scholljegerdes, Eric

Setyabrata, Derico

Shannon, Marcia

Shearer, Jan

Silva, Luis

Silva, Severiano

Silveira Mesquita, Fernando

Simitzis, Panagiotis

Souza, Anaiane

Spangler, Matthew

Sponchiado, Mariana

Sprinkle, James

Steele, Michael

Stein, Hans

Suárez-Trujillo, Aridany

Swanson, Kendall

Tarte, Rodrigo

Teixeira, Izabelle

Tellez-Isaias, Guillermo

Thomas, Milton

Thomson, Jennifer

Tokach, Mike

Tolleson, Douglas

Traverso, Sandra Davi

Tuell, Jacob

Ungerfeld, Rodolfo

Urriola, Pedro

Vargas, Julián

Vedovatto, Marcelo

Vendramini, Joao

Villot, Clothilde

Vinyard, James

Voemesse, Kokou

Walker, John

Warren, Lori

Weber, Thomas

Weimer, Paul

Willmore, Carmen

Wilson, Blake

Wilson, Matthew

Woodworth, Jason

Wu, Fangzhou

Wu, Guoyao

Wulf, Duane

Wyffels, Samuel

Yi, Hongbo

Yin, Jie

Zhang, Gang

Zhang, Shuai

Zimpel, Roney

Zinn, Richard

Zinn, Steven

